# Structural insights into the HBV receptor and bile acid transporter NTCP

**DOI:** 10.1038/s41586-022-04857-0

**Published:** 2022-05-17

**Authors:** Jae-Hyun Park, Masashi Iwamoto, Ji-Hye Yun, Tomomi Uchikubo-Kamo, Donghwan Son, Zeyu Jin, Hisashi Yoshida, Mio Ohki, Naito Ishimoto, Kenji Mizutani, Mizuki Oshima, Masamichi Muramatsu, Takaji Wakita, Mikako Shirouzu, Kehong Liu, Tomoko Uemura, Norimichi Nomura, So Iwata, Koichi Watashi, Jeremy R. H. Tame, Tomohiro Nishizawa, Weontae Lee, Sam-Yong Park

**Affiliations:** 1 Drug Design Laboratory, Graduate School of Medical Life Science, Yokohama City University, Yokohama, Japan; 2 Department of Virology II, National Institute of Infectious Diseases, Tokyo, Japan; 3 Department of Biochemistry, College of Life Science and Biotechnology, Yonsei University, Seoul, South Korea; 4 PCG-Biotech, Seoul, South Korea; 5 Laboratory for Protein Functional and Structural Biology, RIKEN Center for Biosystems Dynamics Research, Yokohama, Japan; 6 Department of Biological Sciences, Tokyo University of Science, Noda, Japan; 7 Department of Cell Biology, Graduate School of Medicine, Kyoto University, Kyoto, Japan; 8 RIKEN SPring-8 Center, Sayo-gun, Japan; 9 Research Center for Drug and Vaccine Development, Tokyo, Japan; 10 Laboratory of Biomembrane Dynamics, Graduate School of Medical Life Science, Yokohama City University, Yokohama, Japan

**Keywords:** Drug discovery, Electron microscopy

## Abstract

Around 250 million people are infected with hepatitis B virus (HBV) worldwide^1^, and 15 million may also carry the satellite virus hepatitis D virus (HDV), which confers even greater risk of severe liver disease^2^. The HBV receptor has been identified as sodium taurocholate co-transporting polypeptide (NTCP), which interacts directly with the first 48 amino acid residues of the *N*-myristoylated N-terminal preS1 domain of the viral large protein^3^. Despite the pressing need for therapeutic agents to counter HBV, the structure of NTCP remains unsolved. This 349-residue protein is closely related to human apical sodium-dependent bile acid transporter (ASBT), another member of the solute carrier family SLC10. Crystal structures have been reported of similar bile acid transporters from bacteria^4,5^, and these models are believed to resemble closely both NTCP and ASBT. Here we have used cryo-electron microscopy to solve the structure of NTCP bound to an antibody, clearly showing that the transporter has no equivalent of the first transmembrane helix found in other SLC10 proteins, and that the N terminus is exposed on the extracellular face. Comparison of our structure with those of related proteins indicates a common mechanism of bile acid transport, but the NTCP structure displays an additional pocket formed by residues that are known to interact with preS1, presenting new opportunities for structure-based drug design.

## Abstract (web summary)

Cryo-electron structures of the hepatitis B virus receptor NTCP show a distinct membrane topology compared with other SLC10 proteins, but a common bile acid transport mechanism that is shared with related mammalian and bacterial proteins.

## Main

Although vaccines are available against HBV, it remains the principal cause of hepatocellular carcinoma, and it is estimated that more than 800,000 people infected with HBV die each year from liver cancer or cirrhosis brought on by HBV and HDV^1^. Treatment with pegylated interferon is often accompanied by adverse reactions, and its efficacy is very limited among patients with HDV^6^. Nucleoside reverse transcriptase inhibitors can be used to control, but not cure, chronic hepatitis arising from HBV^7^, leaving an urgent clinical demand for effective ways to eliminate HBV from the body. The 3.2-kilobase DNA genome of HBV encodes three envelope proteins, called small, middle and large (L). HDV has identical envelope proteins, and is dependent on HBV for their production. Both viruses interact with hepatocytes through the myristoylated N-terminal preS1 domain of the viral L protein. Binding of this domain to the bile salt uptake protein NTCP on the hepatocyte surface induces internalization and infection through a mechanism that is poorly understood^8^. Blocking this process is an attractive means of controlling both HBV and HDV, and myrcludex B^9^, a synthetic analogue of the preS1 lipopeptide has been approved to treat HDV in Europe and Russia. In vitro screening assays have been used to identify other chemical entities that prevent HBV entry into hepatocytes^10,11^. Similar to myrcludex B, these compounds tend to inhibit the native function of the receptor in the uptake of bile salts. Geyer and colleagues have therefore searched for orally available compounds that prevent NTCP from binding to preS1 but permit bile salt uptake^12^. Although such studies have identified a number of promising compounds, a detailed molecular understanding of the binding of both HBV and bile salts to NTCP would help further to guide structure-based drug design. The differences in the sequences between NTCP and related proteins with detailed structural models are too large to enable reliable topology models to be built—we therefore set out to determine the structure of the protein experimentally.

## Overall structure of NTCP

**Fig. 1:**
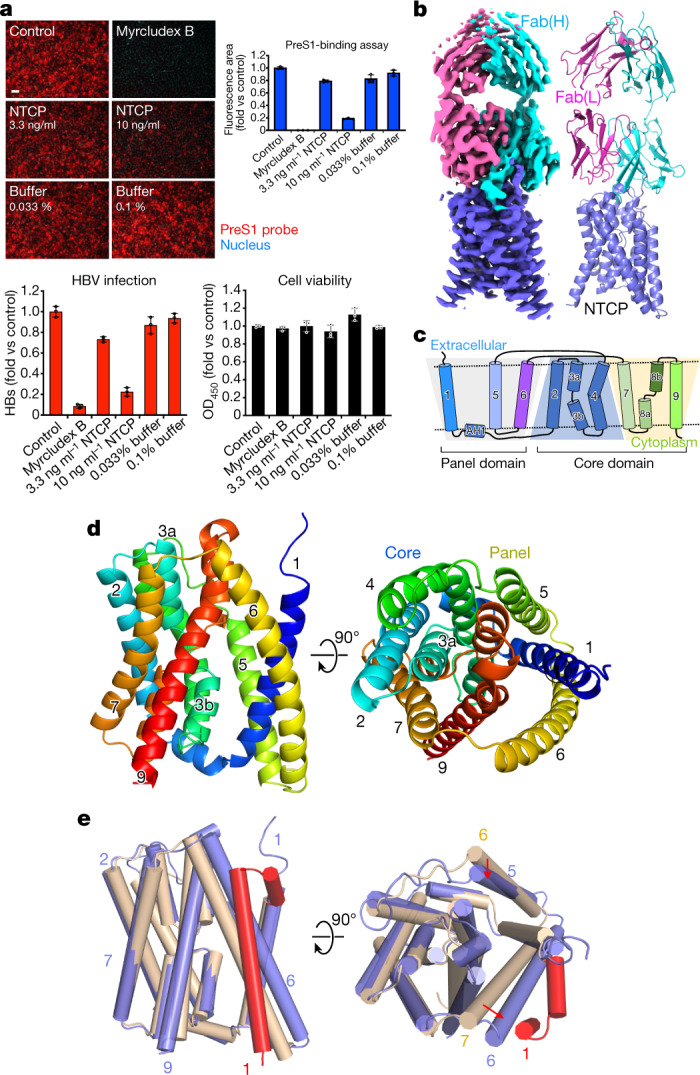
Function and cryo-EM structure of NTCP as HBV receptor. **a**, Functional activity assays of rhNTCP. HBV preS1 attachment and HBV infection were evaluated in HepG2 cells expressing rhNTCP by treating the cells with a preS1 probe or HBV in the presence of 3.3 or 10 ng ml rhNTCP, or 500 nM myrcludex B. Percentages indicated the concentration of DDM in the buffer. Cell viability was measured by MTT assay. Data are mean ± s.d. of three independent samples. Scale bar, 100 µm. HBs, HBV surface protein. **b**, Cryo-EM map contoured with a threshold of 0.020 (left) and ribbon representation (right) of the human NTCP–Fab complex structure. The light and heavy chains of Fab YN69083 are shown in pink and cyan, respectively; NTCP is shown in blue. **c**, Transmembrane topology diagram of NTCP. The transmembrane helices are grouped by functional domain. Shaded trapezoid regions indicate pseudo-symmetrical regions of the protein. Black dashed lines indicate the position of the membrane bilayer. **d**, Two orthogonal views of NTCP shown as a Cα ribbon, with helices numbered from the N terminus. Left, the external face is shown at the top. Right, the protein is viewed from outside the cell. **e**, Comparison of NTCP with the bacterial homologue *Y. frederiksenii* ASBT. NTCP (blue) is superimposed on *Y. frederiksenii* ASBT (wheat) (PDB 4N7X), viewed from two perpendicular directions. The additional N-terminal helix of *Y. frederiksenii* ASBT, which has no equivalent in NTCP, is highlighted in red.

**Extended Data Fig. 1:**
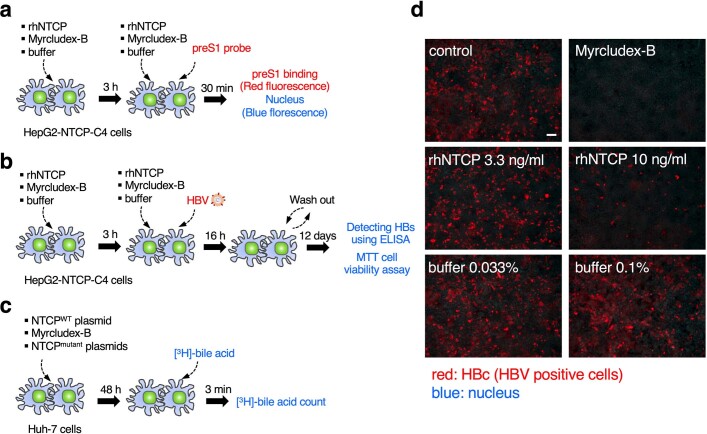
Scheme of assays and fluorescence images of HBV infection assay for recombinant human NTCP (rhNTCP). **a**, Scheme of HBV infection and MTT cell viability assay for rhNTCP. Free rhNTCP competes for ligands with NTCP expressed on the surface of HepG2-NTCP-C4 cells, so that if more free rhNTCP is added, the HBV infection rate of the cells falls. Myrcludex-B, an inhibitor of HBV infection, was used as a positive control. MTT assay was also performed with the same scheme of HBV assay to determine whether additives such as rhNTCP or Myrcludex-B affect HepG2-NTCP-C4 cell viability. **b**, Scheme of HBV preS1 attachment assay for rhNTCP. HepG2-NTCP-C4 cells to which preS1 is attached show red fluorescence by the TARMA-preS1 probe, and HepG2-NTCP-C4 cells to which preS1 is not attached show blue fluorescence around the nucleus. Less preS1 attaches to HepG2-NTCP-C4 cells as more rhNTCP is added. **c**, Scheme of bile acid uptake assay for NTCP. Bile acid uptake was measured in Huh7 cells transfected with the expression plasmid for the wild-type or mutant NTCP. Myrcludex-B was used as a positive control. **d**, Fluorescence images of HBV infection assay in HepG2-hNTCP-C4 cells. It was confirmed that the HBV infection of HepG2-NTCP-C4 cells decreased as more rhNTCP was added. Fluorescent signals for HBc protein were observed with a fluorescence microscope (BZ-X710, KEYENCE). The micrographs shown were obtained in one of three duplicate experiments, which together with the result of Fig. 1a, support the conclusion that rhNTCP inhibits HBV infection. The scale bar in the control image is 100 µm long.

**Extended Data Fig. 2:**
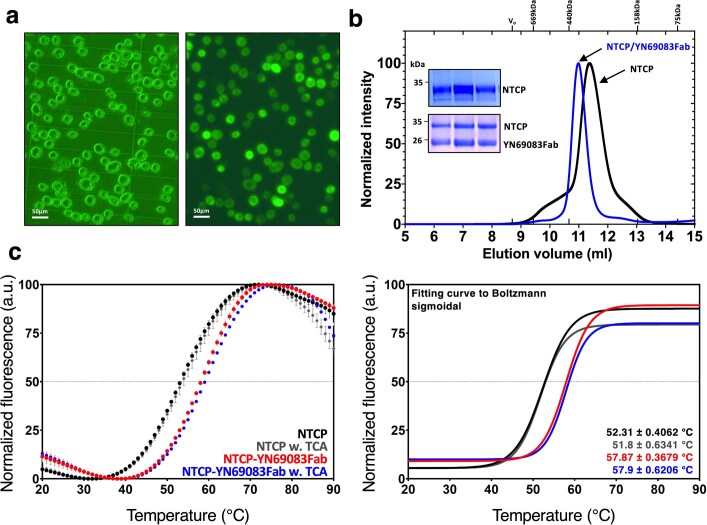
Expression, purification, and biochemical characterization of NTCP and complex with Fab(YN69083). **a**, Phase contrast (left) and fluorescence microscopy image (right) of Sf9 cells expressing NTCP-GFP. Fluorescence indicates level of NTCP-GFP expression. Similar results were obtained in five separate experiments. **b**, Representative size-exclusion chromatography profile of NTCP (black line) and complex with Fab (blue line). Inset shows SDS–PAGE gel of NTCP and complex with Fab used for cryo-EM grid preparation. SDS-PAGE gels were visualized by Coomassie-blue staining. **c**, DSF thermal stability assay of NTCP, and NTCP-Fab complex with or without 50 µM TCA. Raw data were plotted with error bars indicating the standard deviations of double samples (left). The T m values were calculated as a graph fitted to the Boltzmann sigmodal equation (right). Binding of one Fab fragment to NTCP show a strong antibody dependent stabilisation of NTCP, raising the melting point from 52.3 ± 0.4 °C to 57.9 ± 0.4 °C.

HepG2 cells do not normally express NTCP. We expressed recombinant human NTCP (rhNTCP) in these cells using a codon-optimised construct based on *SLC10A1* (the human gene that encodes NTCP) to determine whether the protein is functionally active at the cell surface. We assayed the attachment of preS1 to the cells as described previously^13^ (Extended Data Fig. 1). As shown in Fig. 1a, the expression of rhNTCP enabled a fluorescently tagged preS1 probe to bind to the cells, and rendered the cells susceptible to infection. Both preS1 binding and viral infection could be blocked by either exogenous rhNTCP or myrcludex B^7^. HBV infection assays (Fig. 1a) showed that expression of rhNTCP rendered the cells susceptible to infection, which could be blocked by either exogenous rhNTCP or myrcludex B. MTT assays indicated that addition of exogenous rhNTCP or myrcludex B was not toxic to the cells. We obtained a high yield of purified NTCP by expressing the protein in Sf9 cells as a fusion with green fluorescent protein (GFP) at the C terminus (Extended Data Fig. 2a). The GFP moiety was removed using HRV3C protease and the purified protein, solubilized using *n*-dodecyl-β-d-maltopyranoside (DDM) and cholesterol hemisuccinate (CHS), was found to be stable, with a melting temperature (*T*_m_) of 52 °C. We raised antibodies against NTCP in mice, and one isolated Fab fragment (clone number YN69083) was shown to increase the *T*_m_ of NTCP by more than 5 °C, a similar stabilization to the one that occurs in the presence of 50 μM taurocholate, indicating that the protein is correctly folded (Extended Data Fig. 2).

**Extended Data Fig. 3:**
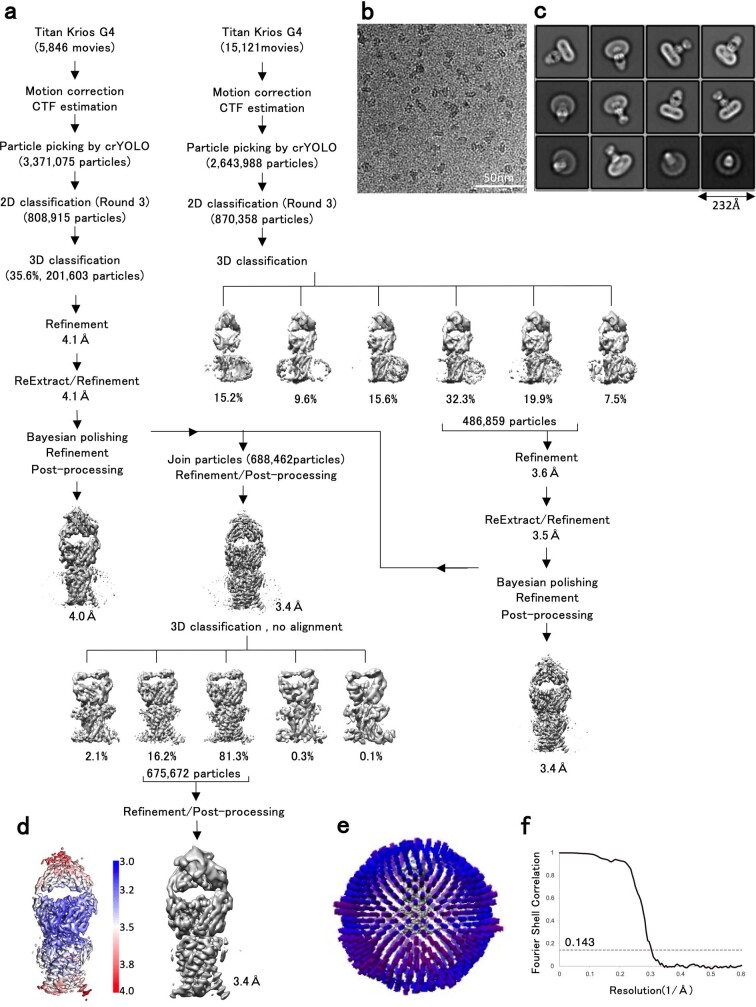
Flow chart of cryo-EM data processing for NTCP-Fab complex. **a**, Schematic flow chart of the classification and refinement procedures to resolve the NTCP-Fab complex structure. **b**, Representative raw micrograph of NTCP-Fab complex. **c**, Gallery of two-dimensional class averages, with a window size of 232 Å. **d**, Final three-dimensional density map coloured by local resolution of the NTCP-Fab complex. **e**, Euler angle distribution of all particles included in the calculation of the final three-dimensional reconstruction. **f**, Fourier shell correlation curve of the globally refined NTCP-Fab complex after the post-processing with RELION. The red line intercepts the y axis at a FSC value of 0.143.

**Extended Data Fig. 4:**
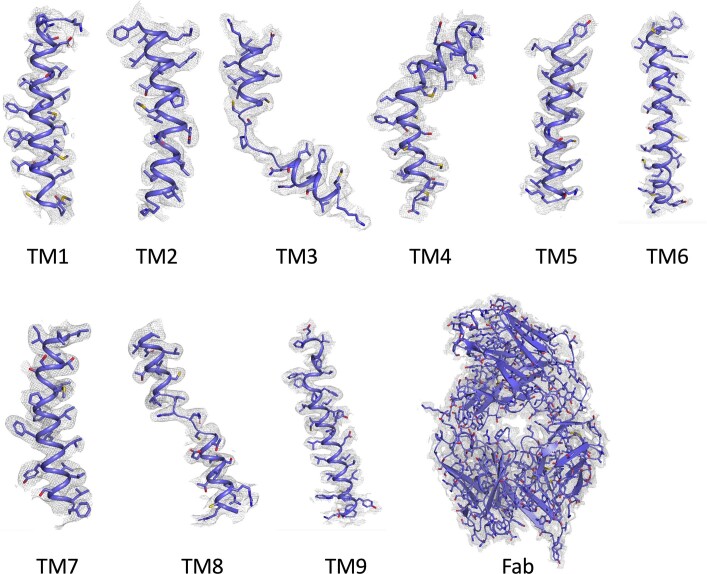
Atomic model and cryo-EM density map of the NTCP–Fab complex. The overall structure of the Fab (bottom right) and magnified views of separate transmembrane helices of NTCP (top and bottom left). The cryo-EM density is shown as a grey mesh with a threshold of 0.020.

We purified the NTCP–Fab complex and determined the structure by cryo-EM (Extended Data Fig. 3 and Extended Data Table 1). The final map, with a resolution of 3.3 Å (Fig. 1b), enabled the construction of a model of almost the entire NTCP protein. Altogether, 297 residues of NTCP (residues 14–310) and 437 residues of the Fab (residues 1–213 of the light chain and residues 1–224 of the heavy chain) were modelled (Extended Data Fig. 4). A schematic topology diagram of the model is shown in Fig. 1c. The model consists of nine transmembrane helices (numbered from TM1 to TM9), equivalent to TM2 to TM10 in the paralogous ASBT proteins from *Neisseria meningitidis* and *Yersinia frederiksenii*. In the bacterial protein models, TM1, TM2, TM6 and TM7 form a ‘panel’ domain, and the remaining transmembrane domains form a ‘core’^4^. Although the panel domain of NTCP comprises only three helices (TM1, TM5 and TM6) and superposes poorly overall on the bacterial ASBTs, the core domain closely matches those of the bacterial ASBTs (Fig. 1d,e).

## PreS1-binding site of NTCP

**Extended Data Fig. 5:**
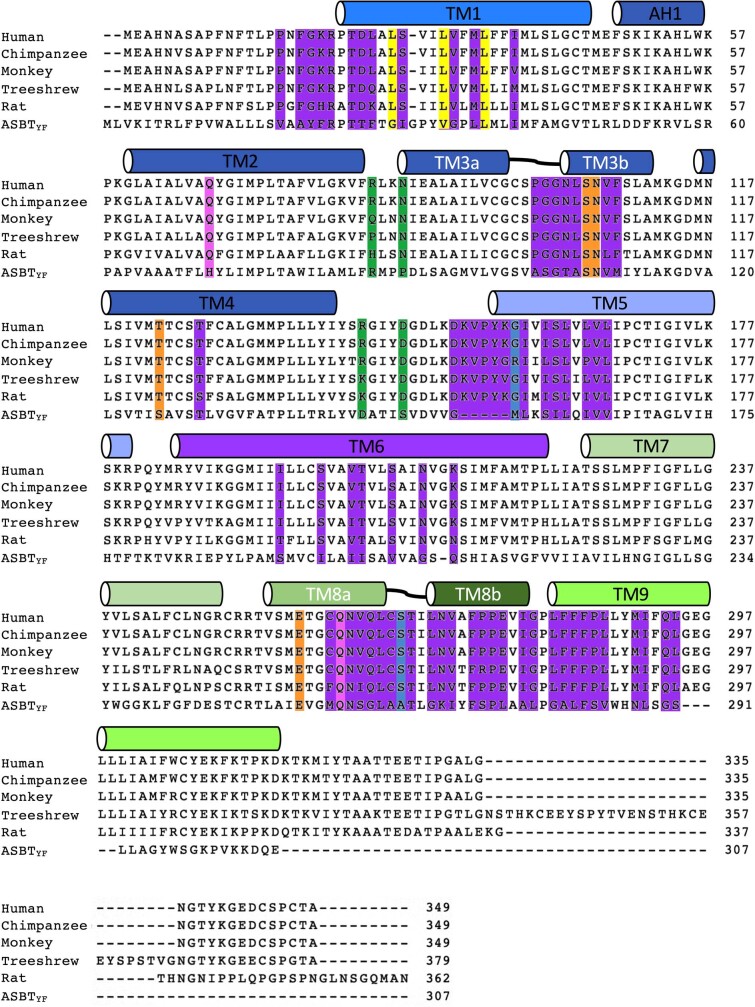
Sequence alignment of the NTCP through the species. Sequence alignment of NTCP of Human, Chimpanzee, Monkey, Treeshrew, Rat and bacterial ASBT, calculated with CLUSTALW. The bars mark the locations of helices in NTCP, and are coloured as in Fig. 1c. Putative residues interacting with Na-1 and Na-2 are highlighted with orange and pink, respectively. Residues forming the TCA / preS1 binding pocket are highlighted in purple. G158 and S267 are highlighted in blue; these residues affect the species specificity of HBV infection among great apes and Old-World monkeys. Other residues known to control species specificity are highlighted in green. Key residues important for TCA and preS1 binding, and NTCP function, are highlighted in yellow.

**Extended Data Fig. 6:**
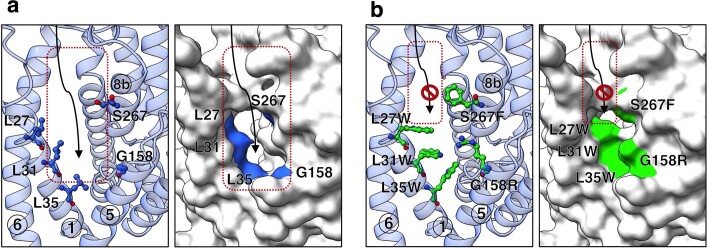
Conformational changes in TCA / preS1 binding pocket according to mutations of key residues that composite pocket. **a**, Cartoon (left) and surface representation (right) of TCA / preS1 binding pocket of wild-type NTCP. Key residues are highlighted as ball-and-stick models and shown in blue. **b**, Cartoon (left) and surface representation (right) of TCA / preS1 binding pocket of quintuple mutant NTCP. The key residues are highlighted in green. Comparison of the binding pocket region of wild-type and quintuple mutant of NTCP. Three leucine residues located in the pathway of TCA transport and G158/S267 residues mentioned in previous studies were selected for mutagenesis. The quintuple mutant structure was obtained by homology modeling (SWISS-MODEL) using the wild-type NTCP structure.

Minor variations in the NTCP of other mammals completely prevent uptake of the virus; human HBV is thus known to infect only two other species, the chimpanzee and the treeshrew^14,15^ (Extended Data Fig. 5). The crab-eating monkey (*Macaca fascicularis*) possesses an NTCP that is 96% identical to the human protein but is unable to support viral entry into cells. A small number of amino acid changes to the monkey protein within a nine-residue motif (from residue 157 to 165) was sufficient to enable preS1 binding and HBV infectivity^3^. These residues at the N-terminal end of TM5 have the sequence KGIVISLVL in human NTCP and GRIILSLVP in *M. fascicularis*. The G158R mutation alone is sufficient to block preS1 binding^16^, presumably by partially filling the cavity between TM1, TM5 and TM8 (Extended Data Fig. 6). Replacing Lys157-Gly158 with Gly157-Arg158 is sufficient to block the binding of the lipopeptide drug myrcludex B^17^ which is based on preS1^18^. Since the binding of preS1 and bile acids is mutually antagonistic^19^, it is highly likely that both ligands interact with residues lining the same cavity.

**Fig. 2:**
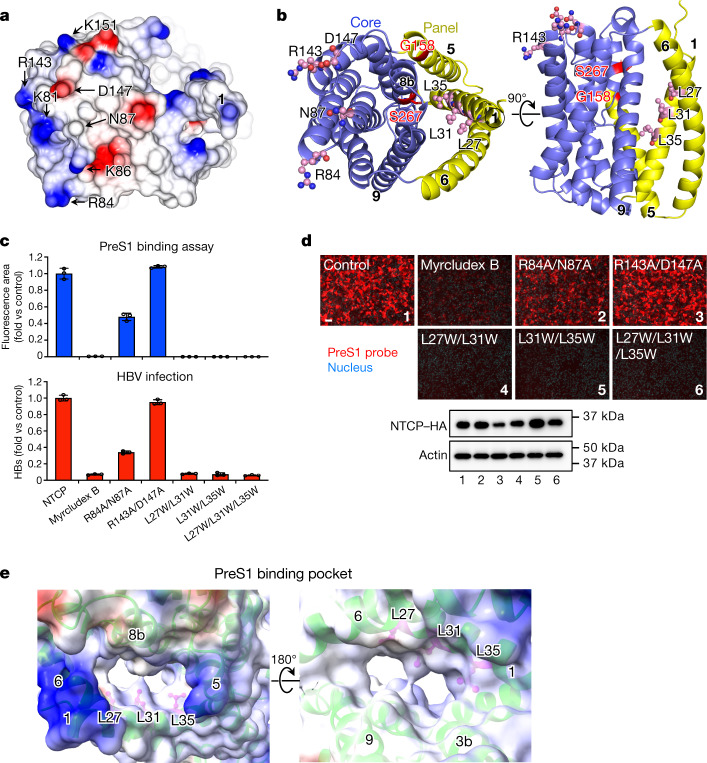
Binding sites for HBV preS1 in human NTCP and mapping of key residues that maintain functionality of NTCP as HBV receptor. **a**, Surface representation of the extracellular face of NTCP, coloured by electrostatic potential. Residues involved in preS1 attachment are labelled. **b**, Ribbon diagrams showing the core (blue) and panel (yellow) domains of NTCP. Left, NTCP is shown in the same orientation as in **a**. The orthogonal view (right) shows the external face at the top. Key residues mutated in this study are shown as ball-and-stick models, coloured by element. G158 and S267 are highlighted in red. **c**, HBV preS1 attachment (from images represented in **d**) and HBV infection were examined using wild-type or mutant NTCP-expressing Huh7 and HepG2 cells, respectively. Myrcludex B (500 nM) was used as a control to inhibit NTCP-mediated HBV preS1 attachment and HBV infection. Data are mean ± s.d. of three independent samples. **d**, Top, fluorescence images of the preS1-mediated HBV attachment assay in Huh7 cells expressing NTCP, showing the effect of NTCP mutations. Bottom, the expression level of NTCPs was monitored by Western blot. Lanes are numbered according to the corresponding images. NTCP and actin (loading control) were run on different gels. Scale bar, 100 µm. **e**, Electrostatic surface potential of the preS1-binding pocket of NTCP. Left, view from the cytoplasmic side. Right, view from the external side. Numbers in circles indicate the transmembrane helices lining the pocket. Leu27, Leu31 and Leu35 are shown as ball-and-stick models in pink. In **a**,**e**, Surfaces with positive and negative charges are coloured in blue and red, respectively, and electrically neutral surfaces are coloured in white.

Replacing only four consecutive residues of mouse NTCP with human NTCP residues 84–87 confers productive binding with the virus^19^. These residues are in an extracellular loop between TM2 and TM3, roughly 30 Å from Gly157, as shown in Fig. 2a. Within this four-residue motif, only Asn87 is common to the human, monkey and treeshrew proteins. None of the residues in this loop region affect bile acid uptake^19^, which suggests the possibility of blocking viral infection without preventing the normal function of NTCP^20^.

**Fig. 3:**
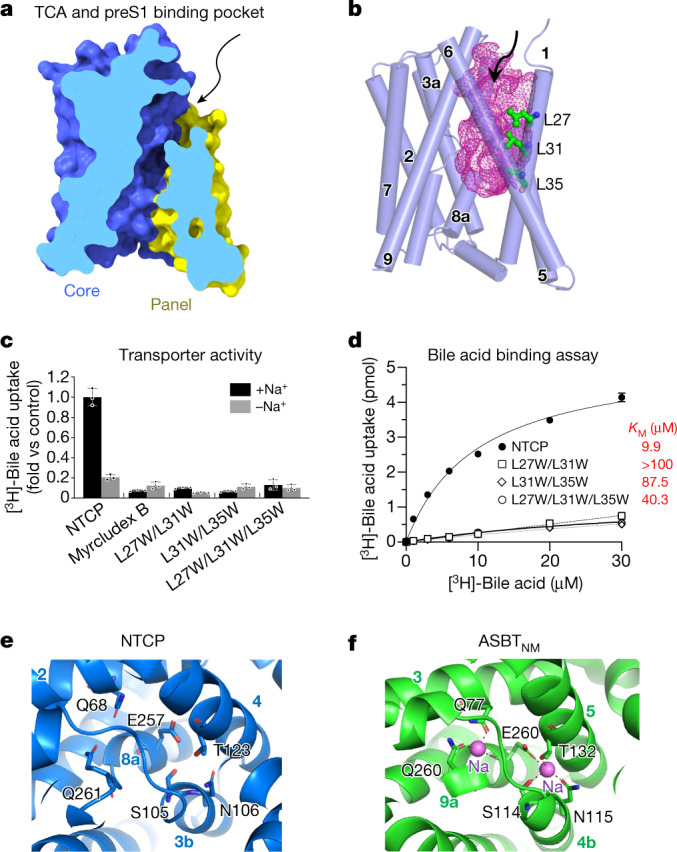
TCA-binding cavity and mutagenesis analysis. **a**, Vertical slice through a surface representation of NTCP, showing the TCA- and preS1-binding pocket or tunnel. The panel and core domains are coloured blue and yellow, respectively. The arrow indicates the external opening of the pocket. **b**, The TCA- and preS1-binding pocket is shown as a pink mesh within the structure of NTCP, which is shown as cylinders, representing helices. Residues Leu27, Leu31 and Leu35 lining the cavity are shown as ball-and-stick models. **c**, Bile acid uptake was measured in Huh7 cells expressing the wild-type NTCP or NTCP mutants, in either sodium-free or sodium-containing buffer at 37 °C for 3 min. The buffer composition is provided in Methods. Myrcludex B-treated wild-type NTCP was used as a control. Mutation of Leu27, Leu31 and Leu35 to Trp blocks TCA transport. Data are mean ± s.d. of three independent samples. **d**, Transport activity in the presence of different concentrations of substrate were used to calculate Michaelis constant (*K*_M_) values by non-linear regression. Data are mean ± s.d. of three independent samples. **e**, Putative sodium ion-binding sites in NTCP. Conserved residues near the crossover are labelled. Helices are labelled with circled numbers. **f**, Sodium ion-binding sites in the previously reported model of *N. meningitidis* ASBT. Sodium ions are shown as spheres.

A single nucleotide polymorphism (SNP) found in about 9% of the population of East Asia yields the NTCP mutant S267F^19,21^. This protein is unable to transport bile acid or support viral infection, suggesting a functional overlap, and the possibility that derivatives of bile acids may prove to be useful leads in drug design^19^. Ser267 is found in TM8, at the extracellular face of the protein (Fig. 2b and Extended Data Fig. 6). It faces TM1 with a 12 Å-wide cavity between them lined by hydrophobic residues including Leu27, Leu31, Leu35 (of TM1) and Leu104 and Val107 of TM3 (Fig. 3a,b). Leu27, Leu31 and Leu35 are conserved only in mammals. Replacing the leucine residues with tryptophan completely blocks both preS1 binding and HBV infection (Fig. 2c, d and Supplementary Fig. 1).

**Extended Data Fig. 7:**
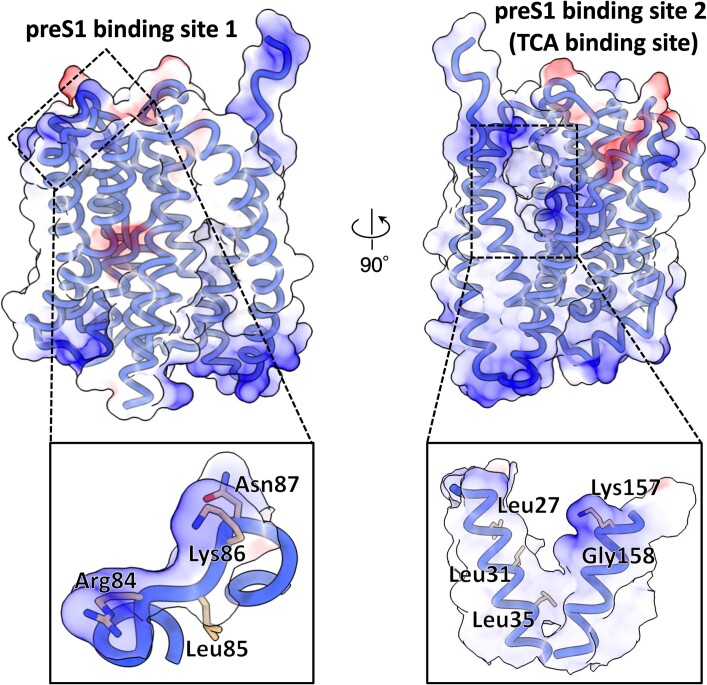
The putative binding sites of preS1 and TCA. The putative binding site of preS1 or TCA are highlighted with dashed boxes. Detailed residues are presented in enlarged boxes below. Surfaces with positive and negative charges are coloured blue and red, respectively, and electrically neutral surfaces are coloured white.

Mapping of residues in preS1 required for HBV infection shows that residues 9–18 are essential, whereas residues 28–39 (and to a lesser extent 39–48) also have a role^22^. The myristoyl group attached to the N terminus of preS1 is believed to sit in the viral lipid envelope, so that residues 2–48 of the protein are presented as a loop at the virion surface. Among human HBV strains, the consensus sequence of preS1 residues 9–18 is NPLGFFPDHQ. Simply replacing Asn9 with lysine blocks infectivity, and the L11K mutation also has a marked effect on binding^3^. PreS1 residues 9–18 may bind to the loop (residues 84–87) of NTCP, with Asn9 of preS1 approaching Asn87 of NTCP, so that Lys9 of the peptide would then repel Lys86 of NTCP (Extended Data Fig. 7).

## Taurocholate transport assay

A sodium gradient across the cell membrane is absolutely required for taurocholic acid (TCA) transport by NTCP (Fig. 3c). TM3 and TM8 of NTCP are both split into two halves, and approach each other closely at this region, called the ‘crossover’. This feature is shared with NhaA^23^, a sodium–proton antiporter^24^, as well as *N. meningitidis* ASBT and *Y. frederiksenii* ASBT^4,5^. Two sodium ion-binding sites (Na-1 and Na-2) have been identified near the crossover in the structure of *N. meningitidis* ASBT^4^, and the side-chains contacting the sodium ions are all preserved in NTCP (Fig. 3e,f). Mutating these sites and their equivalents in NTCP and ASBT results in similar functional impairment, strongly suggesting that their structure and function are conserved. By replacing a glutamate residue (Glu254) at one of these sites with alanine in *Y. frederiksenii* ASBT it proved possible to crystallize the protein in a different conformation from the wild type (Supplementary Fig. 1). The core and panel domains remain largely rigid, but their relative orientations change to expose the substrate-binding sites (for bile acid and sodium ions) to the extracellular or intracellular face of the protein^5^, enabling the gradient of sodium ions to drive uptake of bile acids. Sequential overlay of the panel and core transmembrane helices shows that the conformational change closely matches a rigid body rotation of 18° and translation of about 1 Å.

**Extended Data Fig. 8:**
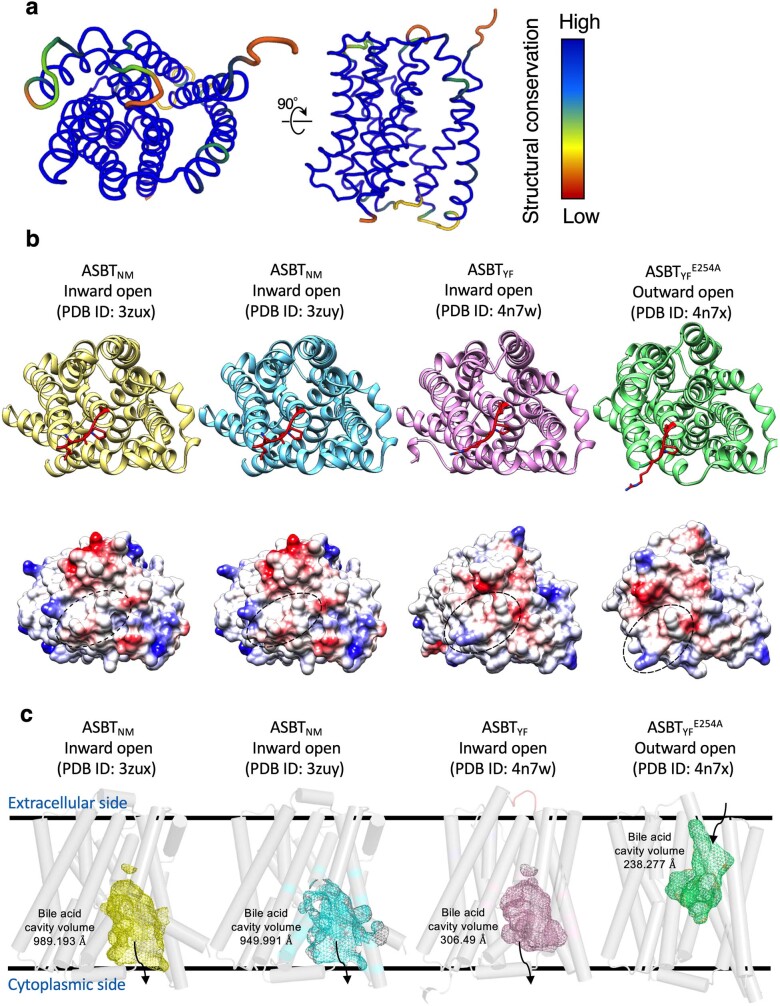
Structural comparison of NTCP with bacterial ASBT homologues. **a**, Structural conservation of NTCP compared with the bacterial ASBT homologues from *Neisseria meningitidis* (ASBT_NM_, PDB ID:3zux, 3zuy) and *Yersinia frederiksenii* (ASBT_YF_, PDB ID:4n7w, 4n7x). Conservation profiles were generated using the Dali web server. **b**, Cartoon (top) and surface charge (bottom) representations of bacterial ASBT homologues in top view. Residues of bacterial ASBT homologues corresponding to 84-87 residues of NTCP were represented by stick model and highlighted as red. In surface charge representations, the regions corresponding to 84-87 loop of NTCP were highlighted as black dashed circle. **c**, The cavities of bile acid were calculated from four different structures of bacterial ASBT homologues. Compared to NTCP, all the bacterial ASBT homologues have small cavities. The volume of the cavities was calculated to be 989 Å^3^, 950 Å^3^, 306 Å^3^ and 238 Å^3^ from left to right, respectively. Black lines indicate membrane boundaries.

Overlaying the NTCP model on models of *Y. frederiksenii* ASBT (wild-type: PDB 4n7w; E254A: PDB 4n7x) suggests that the structure is held in an outward-open conformation more like the mutant *Y. frederiksenii* ASBT (Fig. 3b and Extended Data Fig. 8c). Comparing human NTCP with *Y. frederiksenii* ASBT(E254A), the conformation of the panel is significantly altered by the absence of one helix (Fig. 1e), but applying the same rigid body movement observed in *Y. frederiksenii* ASBT to NTCP gives a rough model of the inward-open form. The C-terminal end of TM1 is pulled away from the core, breaking hydrophobic contacts with TM3 and TM4 near Na-1, and exposing both sodium sites to the cytoplasm. The absence of a helix equivalent to TM1 of ASBT increases the surface exposure of the C-terminal helix of NTCP, but the key interactions controlling the allosteric switch appear to involve Ile38, Met39, Leu42 and Met46 of TM1 (Fig. 3a and Supplementary Fig. 1). Mutation of Met46 to alanine, for example, is expected to disfavour the outside-open form. As mentioned above, the model of NTCP presents a highly apolar pocket to the cell surface, lined by Leu27, Leu31 and Leu35 of TM1 (Figs. 2e and 3a,b). Replacing these leucines with tryptophan not only blocks preS1 binding, but also completely inhibits TCA transport (Fig. 3c,d). It seems likely that the myristoyl group of preS1 can partition from the viral membrane into the bile acid-binding pocket to achieve a firm hold on the protein (Extended Data Fig. 7). Purification of NTCP removes associated lipid molecules, but our model explains the known behaviour of the protein without the need for specific interactions with any cofactor.

## Antibody binding to NTCP

**Fig. 4:**
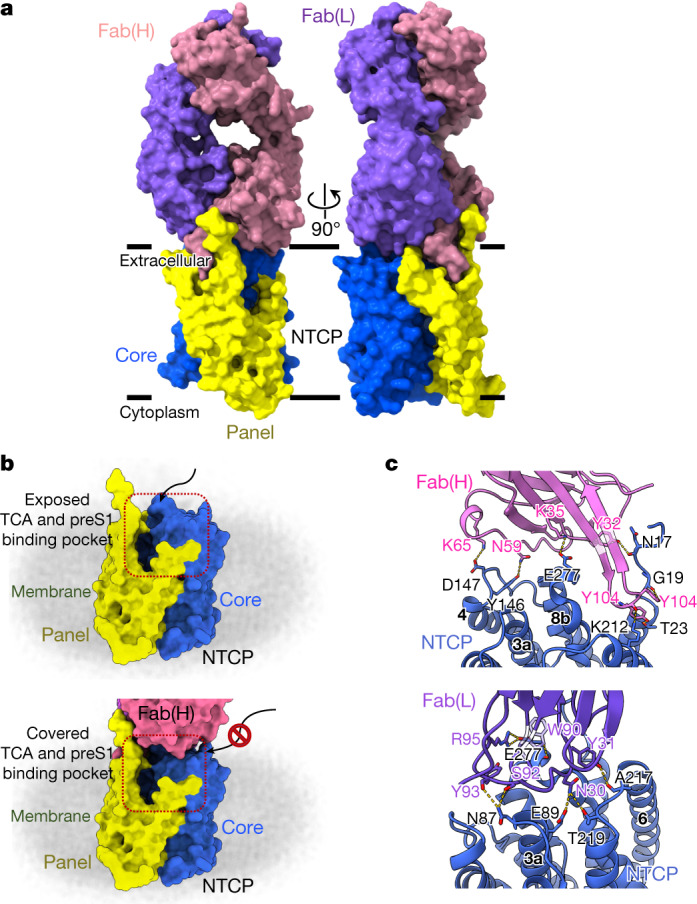
Structural basis of NTCP recognition by Fab YN69083. **a**, Orthogonal views of the NTCP–Fab YN69083 complex shown as molecular surfaces, separately coloured, of each Fab chain and NTCP domain. The intimate contact between the heavy chain and the panel domain is apparent. **b**, Blocking the TCA- and preS1-binding pocket by Fab YN69083. Top, NTCP alone, with the TCA- and preS1-binding pocket exposed on the extracellular face. Bottom, the Fab complex completely occludes the binding pocket, blocking entry of ligands from outside the cell. **c**, Details of the interaction between NTCP and Fab YN69083. Residues forming contacts are shown as stick models and labelled. Blue, NTCP; pink, Fab YN69083 heavy chain; purple, Fab YN69083 light chain. Intermolecular hydrogen bonds are shown as dashed yellow lines.

Antibodies to the N-terminal region of preS1 can block HBV infection^22^, and vaccines were developed decades ago using this and other HBV proteins long before the identity of the receptor was known or precisely which viral proteins it bound^25^. Our model shows in atomic detail the interaction between NTCP and the antibody that we have raised against it (Fig. 4a and Extended Data Table 2). Roughly 950 Å^2^ of the surface area of NTCP is buried on formation of the Fab complex. There are two clusters of interactions, centred around Glu277 of the receptor or Tyr104 of the Fab heavy chain. Glu277 of NTCP—at the loop between TM8 and TM9—makes hydrogen bonds with the side-chain of Arg95 of the light chain, whereas its carbonyl oxygen receives a hydrogen bond from the side-chain of Trp90. At the same time, the indole ring of the tryptophan lies against the NTCP Glu277 side-chain. About 15 Å away, Tyr104 of the heavy chain sits close to Asp24 and Asn209, and forms hydrophobic interactions with the side-chains of Lys20, Phe283 and Phe284 (Fig. 4c). By filling a pocket between the N-terminal regions of TM1 and TM9, Tyr104 of the heavy chain completely prevents movement of the panel domain relative to the core, as envisaged above, since this closes the pocket (Fig. 4b). The antibody therefore appears to be strictly specific for the outward-open form of NTCP. The light chain of the antibody also approaches Asn87 of the TM2–TM3 loop, which helps define species specificity of the virus, but there are no polar contacts or other strong NTCP–Fab interactions in this region to help with modelling preS1.

The NTCP–Fab complex offers several insights that may lead to novel therapeutic treatments for HBV. The first of these is the loop from Asp102 to Val106 of the heavy chain, which engages closely with NTCP through Tyr104. This short region appears to be an excellent drug lead, and suggests that small-molecule mimics could exploit the same pocket and nearby apolar protein surface. Such molecules would necessarily block NTCP function, however, and their principal advantage over myrcludex B would be a lack of antigenic response. To prevent productive HBV interaction with NTCP while permitting bile acid uptake, a more successful approach may be to target the exposed hydrophobic patch between Asn87 and Glu277, which includes Ile88, Pro281 and Leu282.

## Conclusion

NTCP and its paralogue ASBT are part of the small SLC10 family of membrane protein transporters, comprising a bundle of transmembrane helices, that import bile acids and related molecules with fairly broad specificity. For this reason, they have received interest as potential carriers of drugs into cells, enabling the liver and intestine to be targeted. NTCP is the target of myrcludex B, which is currently entering the market as a first-in-class inhibitor of HDV infection, but which suffers from the drawback of preventing normal reuptake of bile acids. This effect may itself be useful to treat obesity or hepatic steatosis^26^. The molecular model of NTCP described here provides detailed information regarding the interaction of the protein with HBV and strong leads to novel means of viral inhibition.

## Methods

### Protein expression and purification

The chemically synthesized codon-optimized *SLC10A1* (UniProt accession number: Q14973) gene was inserted into a modified pFastBacHT-B vector with a haemagglutinin (HA) signal sequence at the N terminus and a HRV3C protease site followed by GFP and a ten-residue His tag at the C terminus. Mutations were produced using overlap extension PCR. For NTCP expression, high-titre viruses were produced with the Bac-to-Bac baculovirus expression system. Sf9 cells were infected at a density of 2 to 3 × 10^6^ cells per ml with high-titre viral stock at 5 times multiplicity of infection (MOI). Twenty micromolar TCA was added 24 h after infection to facilitate the expression of NTCP. Infected cells were further cultured for 36–48 h at 27 °C and then collected. The cells were washed with PSB to remove traces of the culture medium, resuspended using a hypotonic buffer (10 mM HEPES (pH 7.5), 10 mM MgCl_2_, and 20 mM KCl) and stored at −80 °C until further use. To purify membrane fractions, washed cells were disrupted in a hypotonic buffer and subsequently in a high osmotic buffer (10 mM HEPES (pH 7.5), 1 M NaCl, 10 mM MgCl_2_, and 20 mM KCl) in the presence of protease inhibitor cocktail (Roche). Purified membranes were solubilized in buffer containing 10 mM HEPES (pH 7.5), 150 mM NaCl, 10 mM MgCl_2_, 20 mM KCl, 1% (w/v) DDM (Anatrace), 0.2% (w/v) CHS (Anatrace) at 4 °C for 2.5 h. Insoluble materials were removed by ultracentrifugation at 264,902*g*, 4 °C for 40 min, and the supernatant was incubated with DARPin-conjugated CNBr-activated Sepharose 4 Fast Flow beads for overnight at 4 °C. The resin was washed with 10 column volumes of washing buffer I containing 50 mM HEPES (pH 7.5), 800 mM NaCl, 5% (v/v) glycerol, 0.05% (w/v) DDM, 0.01% (w/v) CHS, and then 10 column volumes of washing buffer II containing 50 mM HEPES (pH 7.5), 150 mM NaCl, and 5% (v/v) glycerol, 0.05% (w/v) DDM, 0.01% (w/v) CHS, and then another 10 column volumes of washing buffer I. For elution of the resin-bound NTCP, HRV3C protease and PNGase F were applied to the resin and incubated gently for 16 h at 4 °C. The purified NTCP was concentrated using an Amicon Ultra centrifugal device with 100 kDa MW cut-off, and then applied to a Superdex 200 10/300 column (GE Healthcare), pre-equilibrated with a buffer containing 50 mM HEPES (pH 7.5), 500 mM NaCl, 0.05% (w/v) DDM, and 0.01% (w/v) CHS. After size-exclusion chromatography (SEC), monomeric fractions of NTCP were pooled.

### Protein thermostability assay

BODIPY FL 1-cystine (BFC) (Invitrogen) was dissolved in dimethyl sulfoxide (DMSO) to a concentration of 10 mM. The 10 mM BFC stock was diluted with deionized water to produce a final BFC stock at a concentration of 20 μM. Nine microlitres of protein sample at a concentration of 2 mg ml^−1^ and 1 μl of 20 μM BFC stock was mixed in a PCR tube. All differential scanning fluorimetry measurements were performed with a CFX96 Touch Deep Well Real-Time PCR Detection System (Bio-Rad). Melting curve experiments were performed and recorded by CFX Maestro Software 2.0 (Bio-Rad) in the FAM channel. The samples were cooled to 4 °C and held at that temperature for 1 min, before increasing the temperature by 1 °C per min up to 100 °C. The melting curve results were then fitted to the Boltzmann sigmoid to determine the melting temperature of the protein sample.

### Antibody generation

All the animal experiments conformed to the guidelines of the Guide for the Care and Use of Laboratory Animals of Japan and were approved by the Kyoto University Animal Experimentation Committee. *Rattus norvegicus* NTCP (UniProt accession number: P26435) has 362 residues. Residues 1 to 316 of this sequence, carrying the Q261A mutation, were expressed using the Sf9-baculovirus system and purified. Mouse monoclonal antibodies against rat NTCP(Q261A) were raised essentially as previously described^27^. In brief, a proteoliposome antigen was prepared by reconstituting purified rat NTCP(Q261A) at high density into phospholipid vesicles consisting of a 10:1 mixture of chicken egg yolk phosphatidylcholine (Avanti Polar Lipids) and the adjuvant lipid A (Sigma-Aldrich).

Female, 6-week-old MRL/lpr mice, maintained between 22 to 26 °C and 40% to 60% humidity under a 12-h light cycle, were immunized with the proteoliposome antigen using 3 injections at 2-week intervals. Antibody-producing hybridoma cell lines were generated using a conventional fusion protocol.

Biotinylated proteoliposomes were prepared by reconstituting rat NTCP(Q261A) with a mixture of egg PC and 1,2-dipalmitoyl-*sn*-glycero-3-phosphoethanolamine-N-(cap biotinyl) (16:0 biotinyl Cap-PE; Avanti), and used as binding targets for conformation-specific antibody selection. The targets were immobilized onto streptavidin-coated microplates (Nunc). Hybridoma clones producing antibodies recognizing conformational epitopes in rat NTCP(Q261A) were selected by an enzyme-linked immunosorbent assay on immobilized biotinylated proteoliposomes (liposome ELISA), enabling positive selection of the antibodies that recognized the native conformation of rat NTCP(Q261A). Additional screening for reduced antibody binding to SDS-denatured rat NTCP(Q261A) was used for negative selection against linear epitope-recognizing antibodies. Stable complex formation between rat NTCP(Q261A) and each antibody clone was checked using fluorescence-detection SEC. A total of 13 monoclonal antibodies were isolated that specifically bind to and stabilize conformational epitopes in rat NTCP(Q261A). Cross-reactivity of the antibodies to human NTCP were further evaluated by SEC, and the Fab from the antibody clone number YN69083 was finally selected for use in the cryo-EM single particle analysis. The sequence of Fab YN69083 was determined via standard 5′-RACE using total RNA isolated from hybridoma cells.

### Cryo-EM grid preparation and data collection

Purified NTCP was mixed with the Fab YN69083 in a 2:3 molar ratio. After 1 h incubation at 4 °C, lauryl maltose neopentyl glycol (LMNG) was added to the NTCP–Fab complex to a final concentration of 0.1%. After 1 h incubation at 4 °C, to remove residual DDM and CHS, the reconstituted NTCP–Fab complex was loaded onto a Superdex 200 10/300 column equilibrated in 50 mM HEPES (pH 7.5), 150 mM NaCl, 0.005% LMNG. Peak fractions containing NTCP–Fab complex were pooled and concentrated to 6 mg ml^−1^ using a Vivaspin concentrator (100 kDa cut-off; GE Healthcare). Four microlitres of the protein was applied onto a glow-discharged Quantifoil R0.6/1 300 mesh holey carbon grid using a Vitrobot Mark IV instrument (Thermo Fisher Scientific) with a blotting force of 0 for 3 s at 100% humidity and 4 °C. After being plunge-frozen in liquid ethane, grids were stored in liquid nitrogen and subjected to cryo-EM data collection and analysis.

### Data acquisition and image processing

Cryo-EM imaging was performed on a Titan Krios G4 (Thermo Fischer Scientific) operated at 300 kV, equipped with a Gatan Quantum-LS Energy Filter (slit width 15 eV) and and a Gatan K3 direct electron detector at a nominal magnification of 105,000× in electron-counting mode, corresponding to a pixel size of 0.83 Å per pixel. In the first dataset, each stack movie was recorded for a total of 15.5 electrons per pixel per second for 2.3 s, resulting in an accumulated exposure of 50.5 e^−^ Å^−2^. In the second dataset, each stack movie was recorded for a total of 9.05 e− per pixel per second for 5 s, resulting in an accumulated exposure of 64 e^−^ Å^−2^. These data were automatically acquired by the image-shift method using the EPU software with a defocus range of −0.8 to −2.0 μm. Some 5,846 movie stacks were acquired in the first dataset and 15,121 were acquired in the second. All image processing was performed with RELION-3.1.1^28^. Dose-fractionated image stacks were subjected to beam-induced motion correction using MotionCor2^29^ and the contrast transfer function parameters were estimated using CTFFIND4^30^. In the first dataset, a total of 3,371,075 particles were picked using crYOLO^31^ from the micrographs and extracted at a pixel size of 1.65 Å. These particles were subjected to several rounds of 2D and 3D classifications. The selected 201,603 particles were then re-extracted at a pixel size of 0.83 Å and subjected to 3D refinement, Bayesian polishing^32^, and subsequent postprocessing of the map improved its global resolution to 4.0 Å, according to the Fourier shell correlation (FSC) = 0.143 criterion^33^. In the second dataset, particles were picked using crYOLO^31^ from the micrographs and 2,643,988 particles were extracted with a data pixel size of 3.28 Å. These particles were subjected to several rounds of 2D and 3D classification. The selected 486,859 particles were then re-extracted at a pixel size of 0.83 Å and subjected to 3D refinement, Bayesian polishing, and subsequent postprocessing of the map improved its global resolution to 3.3 Å, according to the FSC = 0.143 criterion.

### Model building and refinement

The initial model of the NTCP–Fab YN69083 complex was built using COOT^34^ to fit models of the *Y. frederiksenii* ASBT(E254A)^5^ (PDB 4N7X) and the antibody fragment Fab structure (PDB 5MYX)^35^ into the cryo-EM map. The high-resolution cryo-EM map allowed side-chain assignments for both NTCP and Fab YN69083 (Extended Data Fig. 4), and shows clear density at the contact region between the two. The entire structure was further manually adjusted and refined using PHENIX^36^ with phenix.real_space refine. The data collection, processing, refinement and validation statistics of the NTCP–Fab YN69083 complex structure are listed in Extended Data Table 1.

### Cell culture

HepG2-NTCP-C4 and HepG2 cells were maintained with DMEM/F-12 + GlutaMax supplemented with 10 mM HEPES, 100 μg ml^−1^ streptomycin, 100 U ml^−1^ penicillin, 10% FBS, 5 µg ml^−1^ insulin and 400 µg ml^−1^ G418^10^. Huh7 cells were cultured in Dulbecco’s modified Eagle’s medium containing 10% FBS, 100 μg ml^−1^ streptomycin, 100 U ml^−1^ penicillin, 100 μM non-essential amino acids, 1 mM sodium pyruvate and 10 mM HEPES^37^.

### Generation of NTCP-expressing cells

HepG2-NTCP-C4 cells stably expressing the human NTCP gene in HepG2 cells were established as described previously^10^. This cell line was used for HBV infection assay, preS1-binding assay and cell viability assay in Fig. 1a. HepG2 and Huh7 cells transiently expressing the wild-type or mutant NTCP were generated by transfection of the corresponding expression plasmids with Lipofectamine 3000 according to the manufacturer’s protocol^13^. These cells were examined to evaluate HBV infection in Fig. 2c, preS1-binding assay in Fig. 2d and NTCP transporter assay in Fig. 3c,d.

### PreS1 binding assay

HBV preS1-mediated attachment to the cells was evaluated by preS1 binding assay as described previously^13^. Cells were cultured with 40 nM C-terminally TAMRA-conjugated and N-terminally myristoylated preS1 peptide, spanning amino acids 2–48 of the preS1 region (preS1 probe), at 37 °C for 30 min and then free preS1 probe was washed away. The cells were then fixed with 4% paraformaldehyde, stained with 4′,6-diamidino-2-phenylindole (DAPI) to observe fluorescence for preS1 probe (red) and the nucleus (blue) by fluorescence microscopy. Exogenous wild-type or mutant rhNTCP in 50 mM HEPES pH 7.5, 500 mM NaCl, 0.03% DDM, 0.006% CHS was added to test blocking of preS1 binding.

### HBV infection assay

HBV inocula derived from the culture supernatant of Hep38.7-Tet cells (genotype D) was prepared as described previously, and used for HBV infection assay^38^. HepG2-NTCP were inoculated with HBV at 4,000 genome equivalent (GEq) per cell in the presence of 4% polyethylene glycol 8000 (PEG8000) for 16 h. After washing away free HBV, the cells were cultured for an additional 12 days and collected to evaluate HBV infection. HBs in the culture supernatant and HBV core protein (HBc) in the cells were detected by ELISA and immunofluorescence, respectively, described previously^39^ and below. Myrcludex B was used as a positive control that has been reported to inhibit HBV infection^39^. Exogenous rhNTCP was added (where used) as in the preS1 binding assay.

### Transporter assay

Transporter activity of NTCP was measured essentially as described^40^. The cells were incubated with [^3^H]-TCA in either sodium-containing buffer (5 mM KCl, 1.1 mM KH_2_PO_4_, 1 mM MgSO_4_, 1.8 mM CaCl_2_, 10 mM d-glucose, 10 mM HEPES, 136 mM NaCl) or sodium-free buffer (5 mM KCl, 1.1 mM KH_2_PO_4_, 1 mM MgSO_4_, 1.8 mM CaCl_2_, 10 mM d-glucose, 10 mM HEPES, 136 mM NMDG) at 37 °C for 3 min to allow [^3^H]-TCA uptake into the cells^41^. After washing to remove free [^3^H]-TCA, the cells were lysed to measure the intracellular radioactivity by a liquid scintillation counter. Figure 3c shows NTCP transporter activity for native and mutant proteins using 20 μM [^3^H]-TCA as substrate. Figure 3d shows NTCP activity in the presence of various concentrations of TCA (0.5, 1, 3, 6, 10, 20 and 30 μM), and the *K*_M_ values for NTCP (wild type), NTCP(L27/31W), NTCP(L31/35W) and NTCP(L27/31/35W) calculated from these data using a previously reported method^42^.

### Cell viability assay

Cell viability was determined by MTT assay performed as described previously^38^.

### Plasmid construction and transfection

NTCP variants were generated by oligonucleotide-directed mutagenesis. The mutated DNA was inserted into the CSII-EF-MCS plasmid (a gift from H. Miyoshi)^43,44^ using XhoI and XbaI sites. Transfection of these plasmids into the cells was performed using Lipofectamine 3000 according to the manufacturer’s protocol^45^.

### Indirect immunofluorescence analysis

Immunofluorescence analysis was conducted essentially as described previously^45^. HBV-infected cells were fixed by treatment with 4% paraformaldehyde and permeabilization with 0.3% Triton X-100. After blocking the cells, they were treated with the primary antibodies against HBc as 1:200 dilution (Thermo Fisher Scientific) and then incubated with Alexa Fluor 594-conjugated secondary antibody as 1:500 dilution and DAPI. HBV-positive cells were observed using fluorescence microscopy.

### Immunoblot analysis

Immunoblot analysis was performed essentially as described previously^38^. Cells were lysed with SDS sample buffer (100 mM Tris-HCl (pH 6.8), 4% SDS, 20% glycerol, 10% 2-mercaptoethanol). The cell lysate was examined using SDS–PAGE and reacted with 1:3,000-diluted anti-HA (Abcam; for NTCP–HA detection) or 1:10,000-diluted anti-actin (Sigma-Aldrich) antibodies as primary antibodies, followed by reaction with horseradish peroxidase (HRP)–conjugated secondary antibodies as 1:3,000 dilution. Samples containing NTCP–HA were treated with 250 U PNGase F to remove *N*-linked oligosaccharides from glycoproteins before analysis by SDS–PAGE^16^. Uncropped SDS–PAGE images are shown in Supplementary Fig. 1.

### Figure preparation

All figures were generated using either UCSF Chimera (v1.15)^46^, UCSF ChimeraX v1.2.1)^47^ or PyMOL (v2.3)^48^. Binding pocket volumes were calculated using CASTp 3.0^49^.

### Reporting summary

Further information on research design is available in the Nature Research Reporting Summary linked to this paper.

## Online content

Any methods, additional references, Nature Research reporting summaries, source data, extended data, supplementary information, acknowledgements, peer review information; details of author contributions and competing interests; and statements of data and code availability are available at 10.1038/s41586-022-04857-0.

### Supplementary information

Supplementary Information This file contains Supplementary Figs. 1–3 and Supplementary Table 1

Reporting Summary

## Figures and Tables

**Extended Data Table 1:**
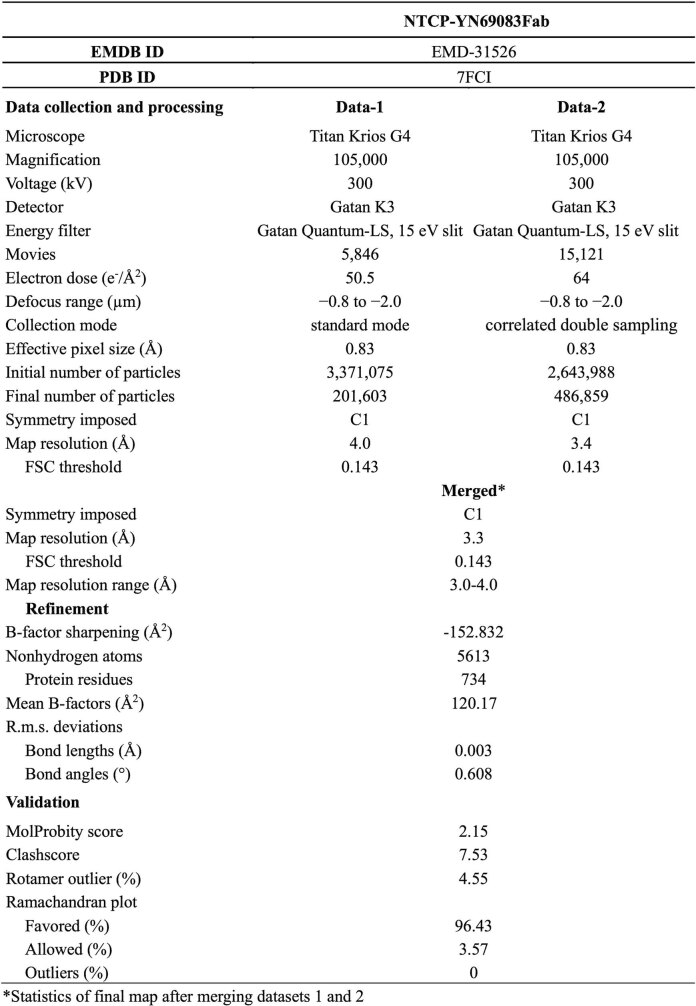
Cryo-EM data collection, refinement, and validation statistics

*Statistics of final map after merging datasets 1 and 2.

**Extended Data Table 2:**
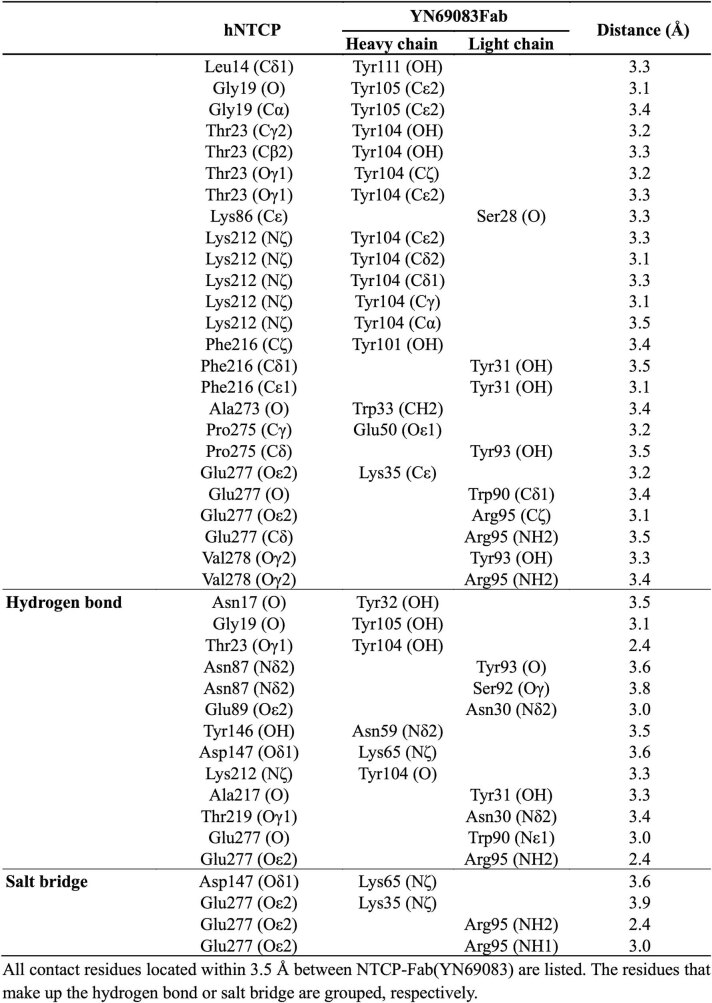
NTCP-Fab(YN69083) contact residues

All contacts less than 3.5 Å between NTCP-Fab(YN69083) are listed. The residues that form hydrogen bonds or salt bridges are grouped.

## Data Availability

The cryo-EM map and the corresponding atomic coordinates have been deposited in the Electron Microscopy Data Bank under accession code EMD-31526 and the Protein Data Bank under accession code 7FCI.
