# Supplementary material for: Structural insights into the HBV receptor and bile acid transporter NTCP
Source: Nature. 2022 May 17;606(7916):1027–31. doi: 10.1038/s41586-022-04857-0 (PMC9242859; doi:10.1038/s41586-022-04857-0)
Supplement: Supplementary file 1 — This file contains Supplementary Figs. 1–3 and Supplementary Table 1 [file 41586_2022_4857_MOESM1_ESM.docx]

**Structural insights into the bile acid transporter NTCP, the receptor for HBV**

Jae-Hyun Park^1^, Masashi Iwamoto^2^, Ji-Hye Yun^3,4^, Tomomi Uchikubo-Kamo^5^, Donghwan Son^3^,

Zeyu Jin^1,3^, Hisashi Yoshida^1^, Mio Ohki^1^, Naito Ishimoto^1^, Kenji Mizutani^1^, Mizuki Oshima^2,6^,

Masamichi Muramatsu^2^, Takaji Wakita^2^, Mikako Shirouzu^5^, Kehong Liu^7^, Tomoko Uemura^7^,

Norimichi Nomura^7^, So Iwata^7,8^, Koichi Watashi^2,6,9^, Jeremy R. H. Tame^1^, Tomohiro Nishizawa^10^,Weontae Lee^3, 4^*, Sam-Yong Park^1^*

**Table of Contents**

Supplementary Figure 1, page 2

Supplementary Figure 2, page 3

Supplementary Figure 3, page 4

Supplementary Table 1, page 7

**Supplementary Figure 1**


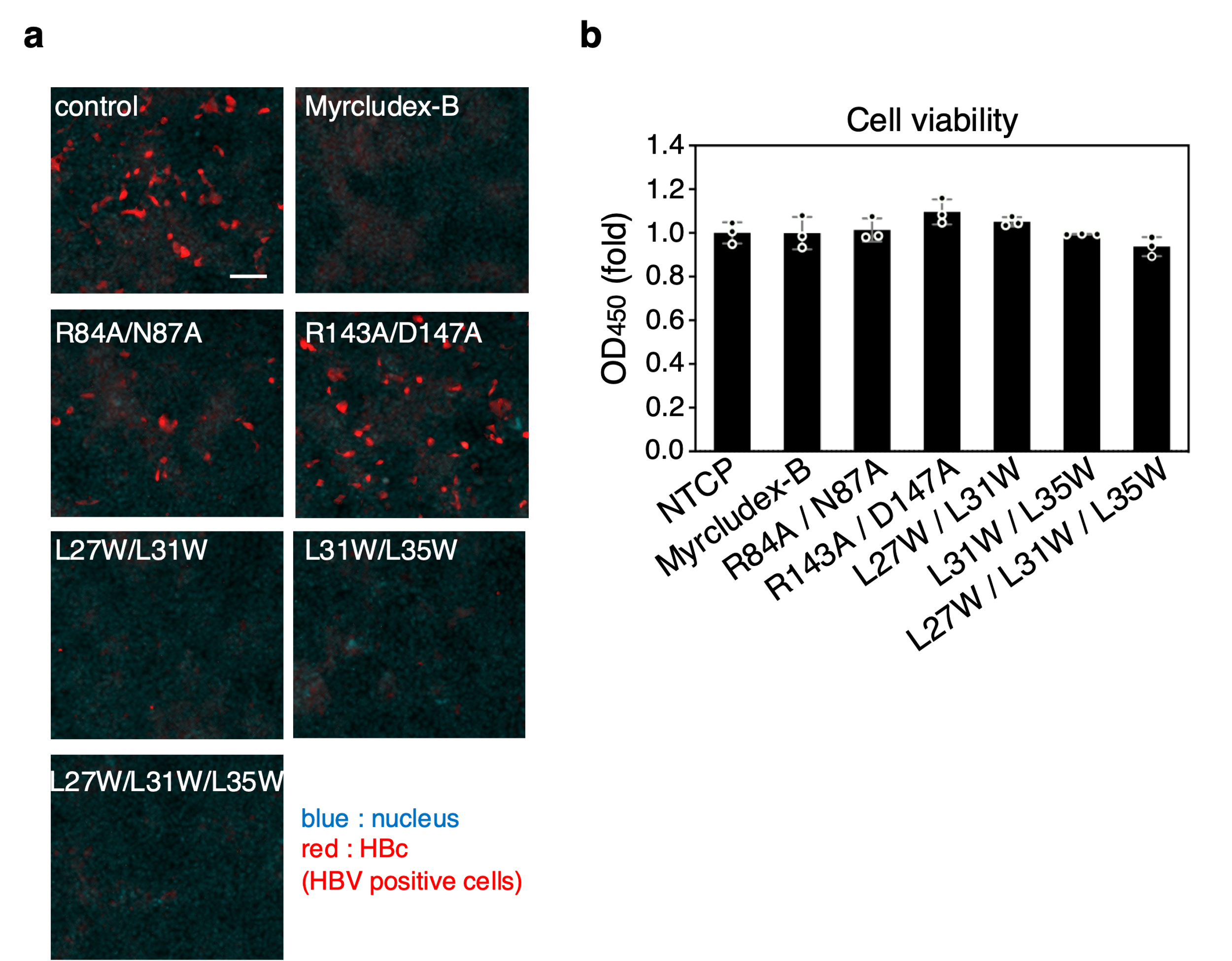


**Supplementary Data Fig. 1 | Fluorescence images used to assess HBV infection via wild-type and mutant NTCP. a,** Fluorescence images showing HBc protein produced in HepG2 cells expressing wild-type NTCP or NTCP mutants, and treated with HBV. It was confirmed that the HBV infection was blocked if Myrcludex-B was added. With NTCPR84A/N87A, the fluorescence signal of HBc decreased compared to the wild-type NTCP control. In the constructs in which leucine residues of NTCP were mutated to tryptophan, almost no fluorescence signal from HBc was observed. Among three separate experiments, these micrographs were obtained in one experiment supporting the conclusion that mutations at L27W/L31W, L31W/L35W, and L27W/L31W/L35W reduced the HBV receptor function of NTCP, together with the results of Fig. 2c and d. The scale bar for micrographs is represented in the control image. Scale bar, 100 µm. **b,** Cell viability measured by MTT assay. The means and standard deviation for three independent samples are shown.

**Supplementary Figure 2**


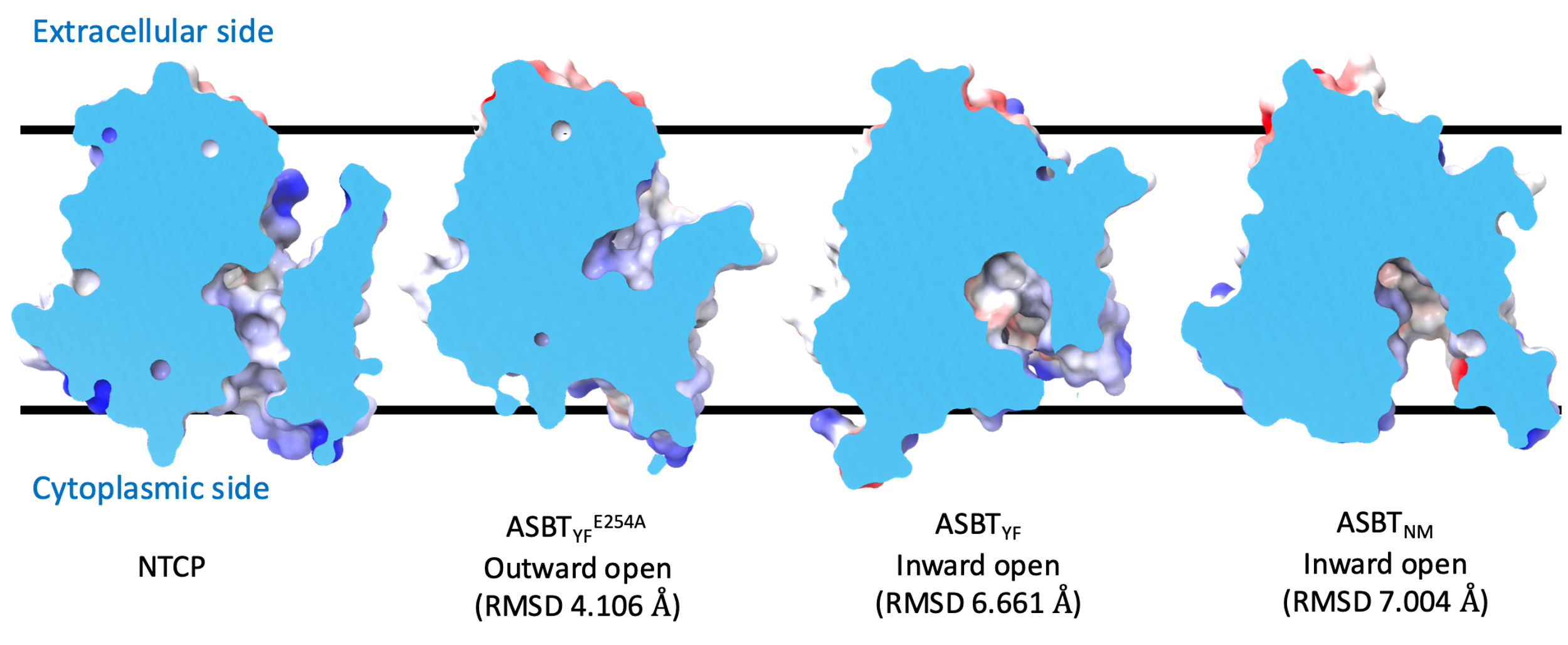


**Supplementary Fig. 2 | Comparison of structural characteristics of TCA / preS1 binding pocket with other bacterial ASBT homologues.** Slices through the molecular surface of NTCP and its bacterial homologues, ASBT_YF_ and ASBT_NM_, revealing the characteristics of the bile acid cavity. Each model shows hydrophobic residues (white) around the binding pocket. The structures of bacterial ASBT homologues are in outward-open or inward-open form, where the cavity is clearly present only extracellular or cytoplasmic side of the cell, whereas in the case of NTCP, the bile acid binding cavity crosses the cell membrane like a tunnel. Black lines indicate membrane boundaries.

**
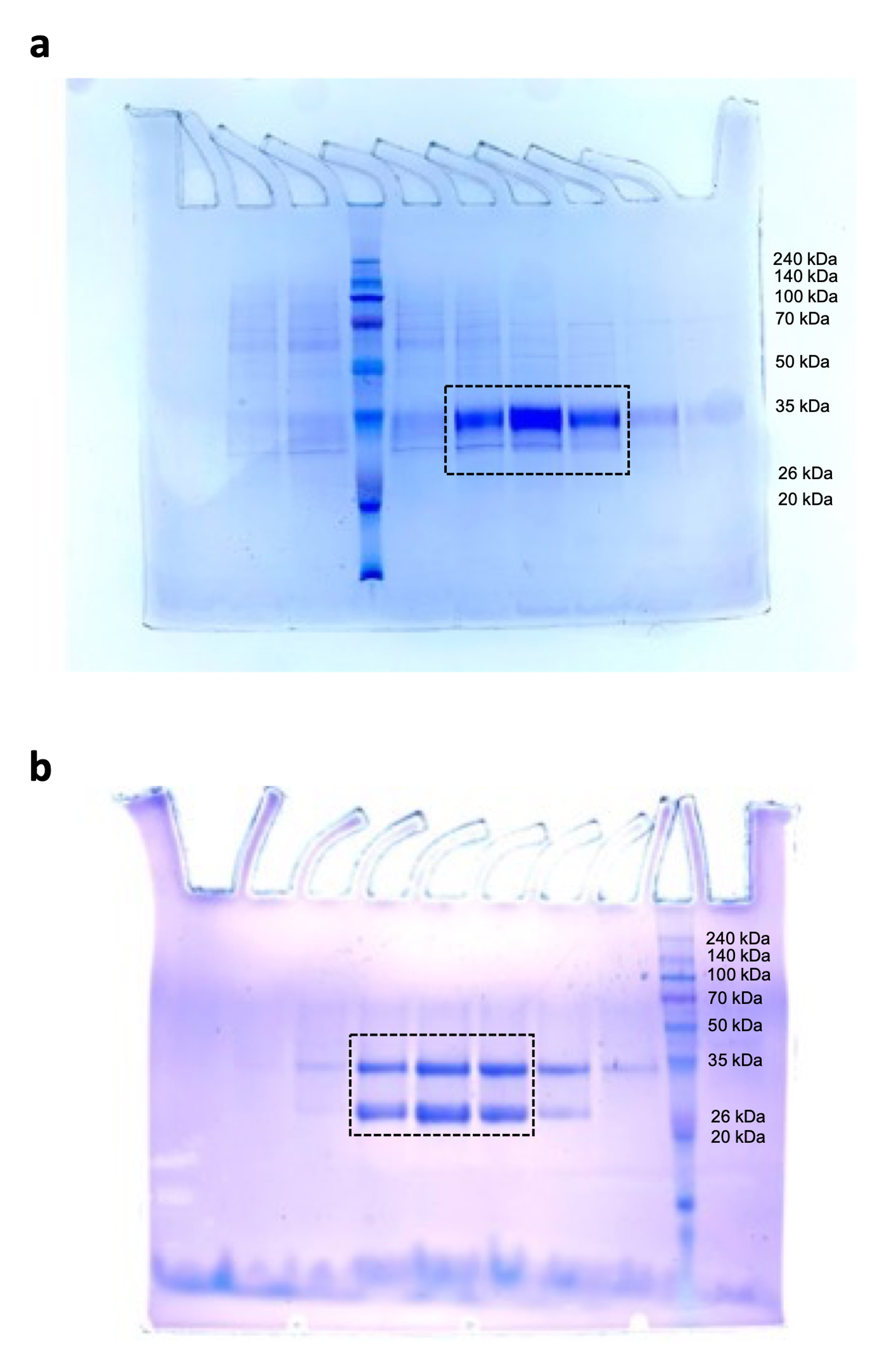
Supplementary Figure 3**

**
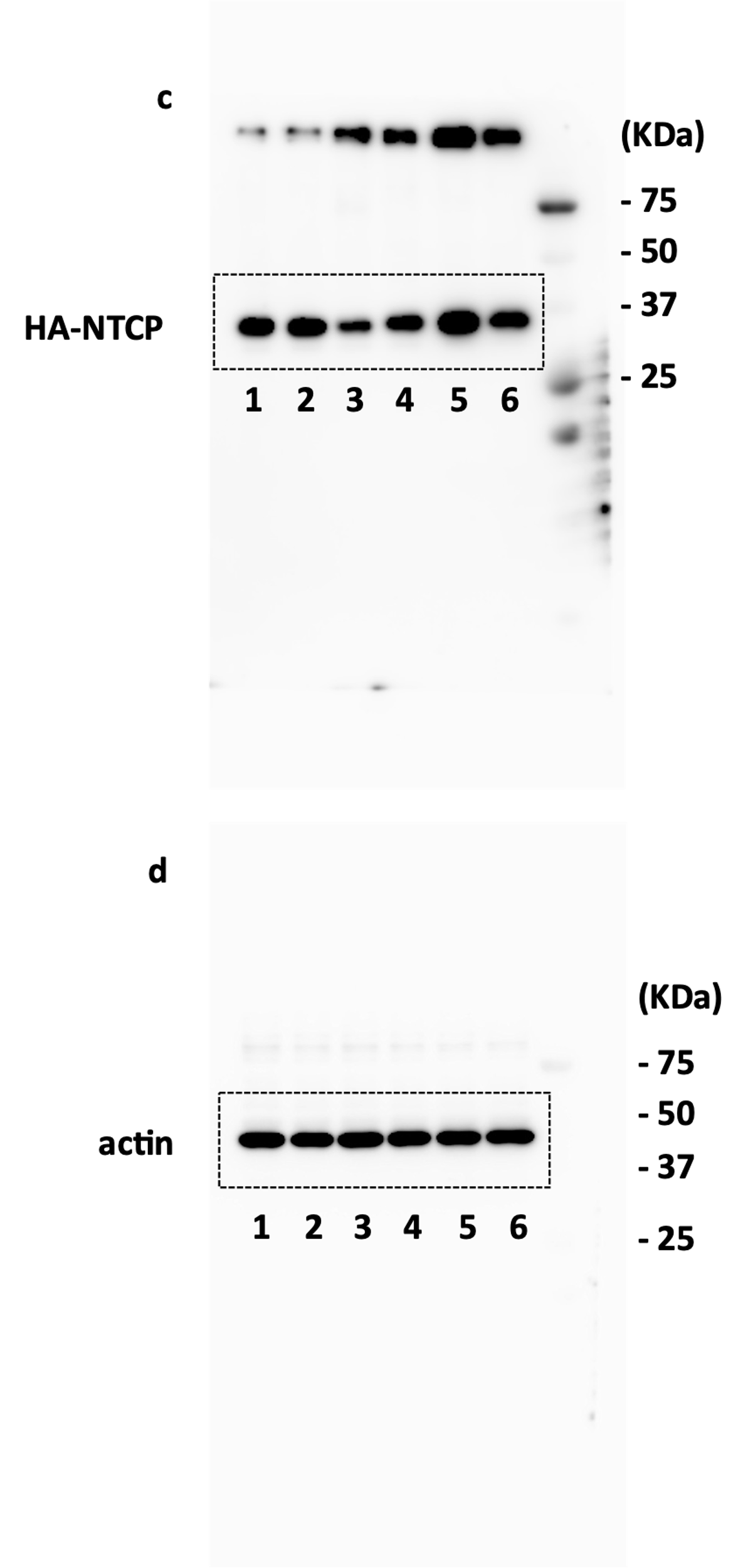
**

**Supplementary Data Fig. 3 | Uncropped images used in this study. a,** SDS-PAGE of size-exclusion chromatography for purified NTCP. This image is used in Extended Data Figure 2b inset (top). **b,** SDS-PAGE of size-exclusion chromatography for NTCP/YN69083Fab complex. This image is used in Extended Data Figure 2b inset (bottom). **c,** Western blot of HA-NTCPs detected using Anti-HA and HRP-linked anti-mouse antibodies as primary and secondary antibody, respectively. **d,** Western blot of actin detected using Anti-actin and HRP-linked anti-mouse antibodies as primary and secondary antibody, respectively. Dotted boxes correspond to samples included in the manuscript.

**Supplementary Table. 1 The amino acid sequence of YN69083 Fab.**

| The light chain  of the YN69083 | DIVMTQSPAIMSASPGQKVTITCSASSSVNYMHWYQQKLGSSPKLWIYDTSKLALGVPARFSGSGSGTSYSLTISSMEAEDAASYFCHQWSSYPRTFGGGTKLEIKRADAAPTVSIFPPSSEQLTSGGASVVCFLNNFYPKDINVKWKIDGSERQNGVLNSWTDQDSKDSTYSMSSTLTLTKDEYERHNSYTCEATHKTSTSPIVKSFNRNEC |
| --- | --- |
| The heavy chain of the YN69083 | EVQLQQPGAELVKPGASVKLSCKTSGYTFTNYWMKWVKQRPGQGLEWIGEINPSNGGTNYNGKFKSKASLTVDKSSSTAYMQLSSLTSEDSAVYYCTILVYDAYYVFAMDYWGLGTSVTVSSAKTTPPSVYPLAPGSAAQTNSMVTLGCLVKGYFPEPVTVTWNSGSLSSGVHTFPAVLQSDLYTLSSSVTVPSSTWPSETVTCNVAHPASSTKVDKKIVPRDC |

The Fab residues interacting with NTCP are underlined.
