# Supplementary material for: Structural insights into the HBV receptor and bile acid transporter NTCP
Source: Nature. 2022 May 17;606(7916):1027–31. doi: 10.1038/s41586-022-04857-0 (PMC9242859; doi:10.1038/s41586-022-04857-0)
Supplement: Supplementary file 2 — Reporting Summary [file 41586_2022_4857_MOESM2_ESM.pdf]

nature portfolio

Corresponding author(s): Sam-Yong Park
Last updated by author(s): Apr 7, 2022

Reporting Summary

Nature Portfolio wishes to improve the reproducibility of the work that we publish. This form provides structure for consistency and transparency in reporting. For further information on Nature Portfolio policies, see our Editorial Policies and the Editorial Policy Checklist.

Statistics

For all statistical analyses, confirm that the following items are present in the figure legend, table legend, main text, or Methods section.

n/a	Confirmed	
☐	☒	The exact sample size (*n*) for each experimental group/condition, given as a discrete number and unit of measurement
☐	☒	A statement on whether measurements were taken from distinct samples or whether the same sample was measured repeatedly
☒	☐	The statistical test(s) used AND whether they are one- or two-sided<br>*Only common tests should be described solely by name; describe more complex techniques in the Methods section.*
☒	☐	A description of all covariates tested
☐	☒	A description of any assumptions or corrections, such as tests of normality and adjustment for multiple comparisons
☒	☐	A full description of the statistical parameters including central tendency (e.g. means) or other basic estimates (e.g. regression coefficient) AND variation (e.g. standard deviation) or associated estimates of uncertainty (e.g. confidence intervals)
☒	☐	For null hypothesis testing, the test statistic (e.g. *F*, *t*, *r*) with confidence intervals, effect sizes, degrees of freedom and *P* value noted<br>*Give P values as exact values whenever suitable.*
☒	☐	For Bayesian analysis, information on the choice of priors and Markov chain Monte Carlo settings
☒	☐	For hierarchical and complex designs, identification of the appropriate level for tests and full reporting of outcomes
☒	☐	Estimates of effect sizes (e.g. Cohen's *d*, Pearson's *r*), indicating how they were calculated

Our web collection on statistics for biologists contains articles on many of the points above.

Software and code

Policy information about availability of computer code

Data collection	EPU 2.9, CFX Maestro Software 2.0
Data analysis	RELION-3.1.1, MotionCor2 1.3.2, CTFFIND4 4.1.9, crYOLO 1.7.6, Phenix 1.19.1, PyMOL 2.3.4, Chimera 1.15, ChimeraX 1.2.5, Coot 0.9.3, GraphPad Prism 9.0.2, BZ-X analyzer version 1.3.0.3 with BZ-H3C software.

For manuscripts utilizing custom algorithms or software that are central to the research but not yet described in published literature, software must be made available to editors and reviewers. We strongly encourage code deposition in a community repository (e.g. GitHub). See the Nature Portfolio guidelines for submitting code & software for further information.

Data

Policy information about availability of data

All manuscripts must include a data availability statement. This statement should provide the following information, where applicable:

- Accession codes, unique identifiers, or web links for publicly available datasets
- A description of any restrictions on data availability
- For clinical datasets or third party data, please ensure that the statement adheres to our policy

The cryo-EM map has been deposited into the Electron Microscopy Data Bank (EMDB) under accession number EMD-31526. The coordinate has been deposited into the Protein Data Bank (PDB) under accession number 7FCI. PDB database (https://www.rcsb.org/) was used in this study to download the coordinates of the ASBT (PDB 4n7x) and the antibody fragment Fab (PDB 5myx)

Field-specific reporting

Please select the one below that is the best fit for your research. If you are not sure, read the appropriate sections before making your selection.

☒ Life sciences ☐ Behavioural & social sciences ☐ Ecological, evolutionary & environmental sciences

For a reference copy of the document with all sections, see nature.com/documents/nr-reporting-summary-flat.pdf

Life sciences study design

All studies must disclose on these points even when the disclosure is negative.

Sample size	Sample sizes were not predetermined for this study. The size of Cryo-EM data were determined by available time of microscope and density of the single particle on grid. No statistical methods used to predetermine sample size for Cryo-EM data.<br>For cell-based assay, three independent experiments (n=3) were performed. A minimum number of experiment was conducted to obtain reliable statistical results in consideration of time and labor power.
Data exclusions	No data were excluded.
Replication	Cell-based experiments and purification of protein were repeated at least three times in independent experiments. Experimental findings were reproduced reliably.<br>Structural analysis described in this study was not repeated. Because structural analysis is not an experiment that should be verified by replication. The result of structural analysis do not change by replication.
Randomization	For Cryo-EM structure determination, the data were randomly divided into two sets by the RELION program.<br>Because no group allocations were performed for cell-based experiments, no randomization was attempted or needed.
Blinding	Investigators were not blinded. No blinding was needed for this study. For both cryoEM structure determination and functional studies, blinding is not necessary because these experiments do not requires subject assessment of the data that may influence the validity of the results.

Reporting for specific materials, systems and methods

We require information from authors about some types of materials, experimental systems and methods used in many studies. Here, indicate whether each material, system or method listed is relevant to your study. If you are not sure if a list item applies to your research, read the appropriate section before selecting a response.

Materials & experimental systems

n/a	Involved in the study	
☐	☒	Antibodies
☐	☒	Eukaryotic cell lines
☒	☐	Palaeontology and archaeology
☐	☒	Animals and other organisms
☒	☐	Human research participants
☒	☐	Clinical data
☒	☐	Dual use research of concern

Methods

n/a	Involved in the study	
☒	☐	ChIP-seq
☒	☐	Flow cytometry
☒	☐	MRI-based neuroimaging

Antibodies

Antibodies used	In house antibody: Fab fragment against NTCP<br>Commercial antibodies: Primary antibody against HBc (Thermo Fisher Scientific; RB-1413-A; rabbit, 1:200 dilution), Alexa Fluor 594-conjugated anti-rabbit secondary antibody (Thermo Fisher Scientific; A-21207; Donkey, 1:500 dilution), Primary antibody against HA for NTCP-HA detection (Abcam; ab49969; mouse, 1:3000 dilution), Primary antibody against actin (Sigma Aldrich; A5441; mouse, 1:10000 dilution), HRP-linked anti-mouse antibody (Cell Signaling Technology; 7076; Horse, 1:3000 dilution).
Validation	The binding of Fab fragment against NTCP was shown by size-exclusion chromatography.<br>Primary antibody against HBc was validated in this study. (Extended Data Fig. 1d control image, we used Alexa Fluor 594-conjugated anti-rabbit antibody as secondary antibody)<br>Primary antibody against HA for NTCP-HA detection was validated by Abcam (Reacts with: Species independent; Suitable for: ELISA, ICC/IF, IP, WB)<br>Primary antibody against actin was validated by Sigma Aldrich (species reactivity<br>pig, Hirudo medicinalis, bovine, rat, canine, feline, human, rabbit, carp, mouse, guinea pig, chicken, sheep;<br>immunohistochemistry (formalin-fixed, paraffin-embedded sections): suitable, indirect ELISA: suitable, indirect immunofluorescence: 1:1,000-1:2,000 using cultured human or chicken fibroblasts, western blot: 1:5,000-1:10,000 using cultured human or chicken fibroblast cell extracts)

Eukaryotic cell lines

Policy information about cell lines

Cell line source(s)	sf9 (Expression Systems, #94-001F), HepG2 (ATCC, HB-8065; kindly gifted from Dr. Tatsuo Miyamura at National Institute of Infectious Diseases), Huh7 (Kindly gifted from Dr. Francis Chisari at The Scripps Research Institute; This cell line is derived from Dr. Francis Chisari's lab.)
Authentication	Commercial cell lines are authenticated by manufacturers. No additional authentications were performed.
Mycoplasma contamination	Not tested for mycoplasma contamination, but there are no indications of mycoplasma contamination.
Commonly misidentified lines (See ICLAC register)	No commonly misidentified cell lines were used.

Animals and other organisms

Policy information about studies involving animals; ARRIVE guidelines recommended for reporting animal research

Laboratory animals	Female, 6 weeks of age MRL/lpr mice for antibody generation against NTCP (maintained at temperature and humidity ranges of 22 to 26 Celsius dgree and 40% to 60%, respectively under a 12-h light, 12-h light cycle.)
Wild animals	This study did not involve wild animals.
Field-collected samples	No filed-collected samples were used.
Ethics oversight	All the animal experiments conformed to the guidelines of the Guide for the Care and Use of Laboratory Animals of Japan and were approved by the Kyoto University Animal Experimentation Committee.

Note that full information on the approval of the study protocol must also be provided in the manuscript.
